# The Association of Cold Ambient Temperature With Fracture Risk and Mortality: National Data From Norway—A Norwegian Epidemiologic Osteoporosis Studies (NOREPOS) Study

**DOI:** 10.1002/jbmr.4628

**Published:** 2022-06-25

**Authors:** Cecilie Dahl, Christian Madsen, Tone Kristin Omsland, Anne‐Johanne Søgaard, Ketil Tunheim, Hein Stigum, Kristin Holvik, Haakon E. Meyer

**Affiliations:** ^1^ University of Oslo, Institute of Health and Society Department of Community Medicine and Global Health Oslo Norway; ^2^ Norwegian Institute of Public Health Department of Health and Inequality Oslo Norway; ^3^ Norwegian Institute of Public Health Department of Physical Health and Ageing Oslo Norway; ^4^ Norwegian Meteorological Institute Oslo Norway

**Keywords:** GENERAL POPULATION STUDIES, FOREARM FRACTURE, HIP FRACTURE, POST–HIP FRACTURE MORTALITY, AMBIENT TEMPERATURE

## Abstract

Norway is an elongated country with large variations in climate and duration of winter season. It is also a high‐risk country for osteoporotic fractures, in particular hip fractures, which cause high mortality. Although most hip fractures occur indoors, there is a higher incidence of both forearm and hip fractures during wintertime, compared with summertime. In a nationwide longitudinal cohort study, we investigated whether cold ambient (outdoor) temperatures could be an underlying cause of this high incidence and mortality. Hospitalized/outpatient forearm fractures (International Classification of Diseases and Related Health Problems, 10th Revision [ICD‐10] code S52) and hospitalized hip fractures (ICD‐10 codes S72.0–S72.2) from 2008 to 2018 were retrieved from the Norwegian Patient Registry. Average monthly ambient temperatures (degrees Celsius, °C) from the years 2008 to 2018 were provided by the Norwegian Meteorological Institute and linked to the residential area of each inhabitant. Poisson models were fitted to estimate the association (incidence rate ratios [IRRs], 95% confidence intervals [CIs]) between temperature and monthly incidence of total number of forearm and hip fractures. Flexible parametric survival models (hazard ratios [HR], 95% CI) were used to estimate the association between temperature and post–hip fracture mortality, taking the population mortality into account. Monthly temperature ranged from −20.2°C to 22.0°C, with a median of −2.0°C in winter and 14.4°C in summer. At low temperatures (<0°C) compared to ≥0°C, there was a 53% higher risk of forearm fracture (95% CI, 51%–55%) and 21% higher risk of hip fracture (95% CI, 19%–22%), adjusting for age, gender, calendar year, urbanization, residential region, elevation, and coastal proximity. When taking the population mortality into account, the post–hip fracture mortality in both men (HR 1.08; 95% CI, 1.02–1.13) and women (HR 1.09; 95% CI, 1.04–1.14) was still higher at cold temperatures. There was a higher risk of forearm and hip fractures, and an excess post–hip fracture mortality at cold ambient temperatures. © 2022 The Authors. *Journal of Bone and Mineral Research* published by Wiley Periodicals LLC on behalf of American Society for Bone and Mineral Research (ASBMR).

## Introduction

Extreme temperatures, in particular cold, have been termed the “underrated risk factor” for many health conditions, and there is a higher winter mortality in several countries.^(^ ^1^ ^)^ Norway is an elongated country in Northern Europe (mainland extending from 58 to 71 degrees north), and with large variations in the duration and degree of winter season.^(^ ^2^ ^)^ It is also a high‐incidence country for osteoporotic fractures, in particular hip fractures, with among the highest rates in the world.^(^ ^3^, ^4^ ^)^ Surges of forearm fractures, the most common osteoporotic fracture, often occur during the winter months, and the high incidence has been attributed to increased precipitation around 0°C outdoor temperature, which results in slippery and icy conditions.^(^ ^5^, ^6^, ^7^, ^8^, ^9^, ^10^ ^)^ Winter peaks in forearm fractures have been found to vary by gender and age. A local study of forearm fracture incidence in Central Norway found seasonal variation in incidence to occur only in women^(^ ^11^ ^)^; however, an older study from Oslo found winter peaks in forearm fracture incidence in both genders and all age groups, except younger men.^(^ ^12^ ^)^ In other European countries and in the United States, findings differ by geographic location, some have found peaks to be more pronounced in men and in the ages <80 years,^(^ ^13^ ^)^ whereas others find peaks in women, which often vary by age.^(^ ^5^, ^6^, ^10^, ^14^, ^15^, ^16^, ^17^ ^)^

The role of ambient temperatures on hip fracture risk is debated, because results have been heterogeneous according to study design, sample size, and temperature parameter used (eg, daily, weekly, mean, or maximum).^(^ ^18^, ^19^ ^)^ Studies from Japan,^(^ ^20^ ^)^ the UK,^(^ ^10^, ^18^, ^21^ ^)^ Spain,^(^ ^22^ ^)^ Canada,^(^ ^23^ ^)^ and Australia^(^ ^24^ ^)^ have found an increase in hip fracture risk with colder temperatures, which differs by gender and age. Although most hip fractures occur in the home,^(^ ^25^, ^26^ ^)^ a higher incidence of hip fractures has been found in winter compared to summer season in Norway.^(^ ^27^ ^)^ The incidence is highest in the south and increases with distance from the milder coastline^(^ ^28^, ^29^ ^)^; conversely, in neighboring Sweden, the incidence increases toward the north.^(^ ^30^ ^)^ Hip fractures are serious injuries, with high excess mortality in older adults; within 1 year after the fracture 20% to 30% of patients have died.^(^ ^31^, ^32^, ^33^ ^)^ One hypothesis is that low temperatures may be a risk factor for inadequate thermoregulation of the body, which in turn may affect the risk of hip fracture and possibly mortality through increased risk of falling, even if staying indoors.^(^ ^34^ ^)^ Cold has long been known to be related to excess winter mortality from all causes,^(^ ^1^ ^)^ but hip fracture specific data are lacking.

With prospective data from the entire Norwegian population over a period of 11 years we aimed to study whether cold ambient temperature (<0°C) was associated with: (i) incidence of forearm fractures, (ii) incidence of hip fractures, or (iii) post–hip fracture mortality rate. This is the first study to use nationwide individual data with meteorological data linked to the residential location of all inhabitants to estimate the independent effect of cold winter temperatures on fracture incidence and postfracture mortality, adjusting for possible regional and individual confounders.

## Patients and Methods

### Study design and study population

For this longitudinal open cohort study we included all individuals over 40 years (forearm fracture outcome) or 50 years (hip fracture and mortality outcome) residing in Norway between January 1, 2008 and December 31, 2018.

### Fractures and Charlson's comorbidity index

Hospitalized hip fractures and hospitalized/outpatient forearm fractures were retrieved from the Norwegian Patient Registry (NPR). The identification of incident hip fractures was based on computerized discharge diagnosis (International Classification of Diseases and Related Health Problems, 10th Revision [ICD‐10] code S72.0–S72.2), relevant Nordic Medico‐Statistical Committee surgical procedure codes (NOMESCO), and the time between hospital admissions for hip fractures (hospital admissions for hip fracture diagnoses <3 weeks apart were counted as the same episode). The procedure for quality assurance was the same as for the NOREPOS hip fracture database (https://norepos.w.uib.no) and hip fracture registrations in the NPR have previously been validated.^(^ ^35^, ^36^, ^37^ ^)^ Incident forearm fractures were defined as registered diagnosis code S52 with all subgroups (ICD‐10), but because incident forearm fractures are not consistently registered with NOMESCO surgical procedure codes, a washout period of 6 months between registrations with S52 codes was applied. Each individual could be registered with up to two hip fractures and multiple forearm fractures (0.4% had more than two fractures) during the observation period. We performed separate analyses for forearm and hip fractures.

Charlson's Comorbidity Index (CCI) was calculated by the NPR based on registered diagnoses within the same year as the fracture,^(^ ^38^ ^)^ and obtained for all hip fracture patients. In correspondence with Quan and colleagues,^(^ ^38^ ^)^ each diagnosis was assigned a weight from 1 to 6 according to the estimated 1‐year mortality hazard ratio from a Cox proportional hazards model, and summarized into a weighted index between 0 (no comorbidity) and 15, thus taking into account the number and the severity of comorbid disease.^(^ ^38^ ^)^

### Temperature and precipitation

Average monthly ambient temperatures (degrees Celsius, °C) and total monthly precipitation (millimeters, mm) were provided by the Norwegian Meteorological institute (MET Norway) using seNorge_2018, a collection of long‐term high‐resolution terrain‐following gridded (1 × 1 km) datasets covering Norway.^(^ ^39^ ^)^ These data are generated using optimal interpolation and are considered a well established source for temperature data in Norway.^(^ ^39^ ^)^ Data from 2008 to 2018 were extracted from seNorge_2018 and were based on 700–1000 daily in situ weather observations combined with a physical model for monthly estimations.^(^ ^39^ ^)^ The temperature estimates were linked to the nearest basic statistical unit (small geographic area) using Geographic Information Systems (GISs) in ArcMap (version 10.7.1). There are about 14,000 basic statistical units in Norway.^(^ ^40^ ^)^

### Demographic data, mortality

Statistics Norway provided individual‐level demographic data for all inhabitants: Gender, age (continuous in years), mortality (on a monthly basis), change in residence (moving, immigration and emigration), education (number of years), marital status (unmarried, married, previously married; ie, separated, divorced or widow/widower) and immigrant status (born in Norway with two Norwegian born parents, immigrant [foreign born with two foreign‐born parents], other [born abroad or in Norway with one Norwegian‐born parent or born abroad with two Norwegian‐born parents]). In addition, we obtained residency data (including region of residence) for each individual and urbanization degree for each municipality. Urban/rural residency was calculated as the number of residents within a municipality residing in densely populated areas, divided by the total number of residents for each year,^(^ ^41^ ^)^ creating a scale from 0.0 to 1.0 indicating increasing degree of urbanization. Residential elevation and distance from the coast (including fjords), both continuous in meters, were calculated for each residence in Norway and averaged within the basic statistical unit using GIS.^(^ ^28^ ^)^ Seasonal variation was assessed by calendar month of fracture.

### Statistics

Ambient temperatures were slightly right‐skewed and are therefore presented as medians (p50) with interquartile ranges (IQRs) across subgroups. Age‐standardized incidence rates and mortality per 10,000 person‐years were calculated using direct standardization and plotted over the temperature span from −15 to 21°C (unstable rates at < −15 and >21°C), using the average age distribution of the population for the total period 2008 to 2018 in 5‐year age groups as standard. To estimate incidence rate ratios (IRRs) with 95% confidence intervals (CIs) of total number of hip and forearm fractures, the number of fractures and person years were aggregated by strata of calendar year, calendar month, gender, health region of residence, education level, marital status, and immigrant status. Average temperature, along with average age, urbanization degree, and geography (elevation and coastal proximity), were calculated within these strata. Poisson models with person years as offset were fitted, adjusting for relevant confounders. Fracture incidence may also vary by other factors (eg, duration of daylight) that are related to season, but not directly due to temperature. In a sensitivity analysis, further adjustment was made for season and precipitation level, both in cubic splines with four knots; however, these were considered to act as mediators (Directed Acyclic Graphs constructed using the Daggity tool,^(^ ^42^ ^)^ Figs. S1 and S2).

In the main analysis, temperature was categorized as <0°C and ≥0°C to study the IRRs of cold winter temperatures. Forearm and hip fracture incidence were also predicted over age, and stratified by temperature and gender using marginal effects. In addition, to study a possible dose–response, temperature was categorized into four groups: <−5°C, −5 to <0°C, 0 to 5°C, >5°C (reference group).

Post–hip fracture mortality by temperature (<0°C versus ≥0°C, at the month of death) was estimated using flexible parametric models (stpm2 command in Stata version 16.1; StataCorp, LLC, College Station, TX, USA), which allow nonproportional hazards, generating hazard ratios (HRs) with 95% CIs. We adjusted for age, urbanization, elevation, coastal proximity, region of residence (North, Central, West, and South‐East), education level, marital status, and immigrant status, and stratified by gender. Estimates were also further stratified on CCI (three groups). In additional analyses, the background mortality was taken into account by merging the hip fracture data with the gender‐ and age‐specific mortality of the total Norwegian population by calendar year (2008–2018) and month (January–December), and estimating relative survival in the flexible parametric models (bhazard option). Survival by temperature was predicted over gender and age in both the hip fracture patient group and the background population.

### Ethics

The study linkages were approved by the Regional Committee for Medical and Health Research Ethics. The University of Oslo performed a Data Protection Impact Assessment (DPIA) in agreement with the General Data Protection Regulation.

## Results

Table 1 shows the number of person‐years and fractures, along with the median ambient temperature in the period 2008 to 2018, by background factors. The monthly temperature in Norway ranged from −20.2°C to 22.0°C, with a median temperature of −2.0°C in winter season (December through February) to 14.4°C in summer season (June through August). The warmest temperatures were recorded in the urban areas (2°C higher than in rural areas) and in the western part of the country (4.5°C higher than in the north, which had the lowest temperature). Temperature varied by residential elevation, with higher temperature at lower elevation closer to the coast, but not by individual factors such as age, gender, and education (Table 1); 0.3% of women and 0.6% of men had missing in ambient temperature, mainly due to missing residential location (basic statistical unit), and were excluded from the analyses.

**Table 1:** Number of Person‐Years, Forearm Fractures (≥40 Years) and Hip Fractures (≥50 Years) with Average Temperature and Nationwide Population (Ages 40–102) in the Years 2008–2018, by Background Characteristics

Characteristic	Person‐years	Forearm fracture (*n*)^a^	Hip fracture (*n*)^b^	Temperature (median °C in years 2008–2018)	IQR^c^
Total	27,193,188	127,794	103,591	5.6	11.4
Age (years)					
40–79	24,839,806	106,413	40,340	5.5	11.4
80–102	2,353,382	21,381	63,251	5.7	11.4
Gender					
Male	13,343,497	29,684	32,540	5.5	11.4
Female	13,849,691	98,110	71,051	5.6	11.4
Education (years)					
<12	12,093,713	69,373	79,876	5.4	11.4
12	5,932,123	20,046	9232	5.6	11.4
>12	8,683,695	36,962	13,734	5.6	11.4
Marital status					
Unmarried	4,588,440	15,932	8874	5.5	11.5
Married	15,391,211	64,271	34,868	5.5	11.4
Previously married^d^	6,838,418	45,569	57,956	5.6	11.4
Immigrant status					
Norwegian^e^	23,578,161	116,031	99,887	5.4	11.4
Foreign^f^	2,730,308	8528	2786	5.7	11.4
Other^g^	884,713	3235	918	5.8	11.5
Season^h^					
Winter	6,788,647	42,552	29,981	−2.0	5.4
Spring	6,839,252	29,893	25,273	4.8	6.4
Summer	6,801,230	28,156	23,534	14.4	2.9
Fall	6,764,059	27,193	24,803	6.4	6.9
Calendar year					
2008–2010	6,967,625	33,087	28,583	5.3	12.1
2016–2018	7,835,903	37,409	27,911	5.3	12.1
Urban/rural^i^					
Rural	32,021	128	266	4.0	11.4
Semirural	15,616,920	72,646	60,912	4.7	11.4
Urban	11,533,104	54,997	42,400	6.1	11.4
Region^j^					
North	2,672,062	11,705	10,216	2.3	11.1
Central	3,785,137	18,907	15,007	5.4	10.1
West	5,397,706	23,119	19,526	6.8	9.4
South‐East	15,331,220	74,047	58,836	5.8	12.6
Residential elevation					
≤108 m^k^	14,604,980	66,441	55,565	5.9	10.7
>108 m^k^	12,576,768	61,330	48,009	4.7	12.8
Coastal proximity					
≤15.7 km^k^	17,341,047	80,937	66,004	5.9	10.8
>15.7 km^k^	9,840,701	46,834	37,570	4.4	13.2
Precipitation					
≤107 mm^k^	15,900,337	75,910	60,814	5.2	12.2
>107 mm^k^	11,281,708	51,861	42,764	6.2	10.8

IQR = interquartile range.

^a^ 40–102 years.

^b^ 50–102 years.

^c^ IQR (75th‐25th percentile).

^d^ Divorced, separated, widow/widower.

^e^ Norwegian born with two Norwegian born parents.

^f^ Foreign born with two foreign born parents.

^g^ Foreign or Norwegian born with one Norwegian parent; foreign born with two Norwegian parents.

^h^ Winter: December, January, February; Spring: March, April, May; Summer: June, July, August; Fall: September, October, November.

^i^ Urbanization degree: 0–0.333 = rural; 0.3331–0.667 = semi‐rural; 0.6671–1 = urban.

^j^ Health Trust areas: North (counties) = Nordland, Troms, Finnmark; Central (counties) = Møre og Romsdal, Trøndelag; West(counties) = Rogaland, Hordaland, Sogn og Fjordane; South‐East (counties) = Østfold, Akershus, Oslo, Buskerud, Hedemark, Oppland, Vestfold, Telemark, Aust‐Agder, Vest‐Agder.

^k^ Mean cutoff.

There was a higher incidence of both forearm and hip fractures at low temperatures (<0°C) compared with temperatures ≥0°C: IRR forearm = 1.53 (95% CI, 1.51–1.55); IRR hip = 1.21 (95% CI, 1.19–1.22), when adjusting for potential confounders: age, gender, calendar year, urbanization level, region of residence, residential elevation, and coastal proximity (results not shown in tables). Other individual factors (education level, immigrant background, and marital status) did not affect the estimates.

**Fig. 1:**
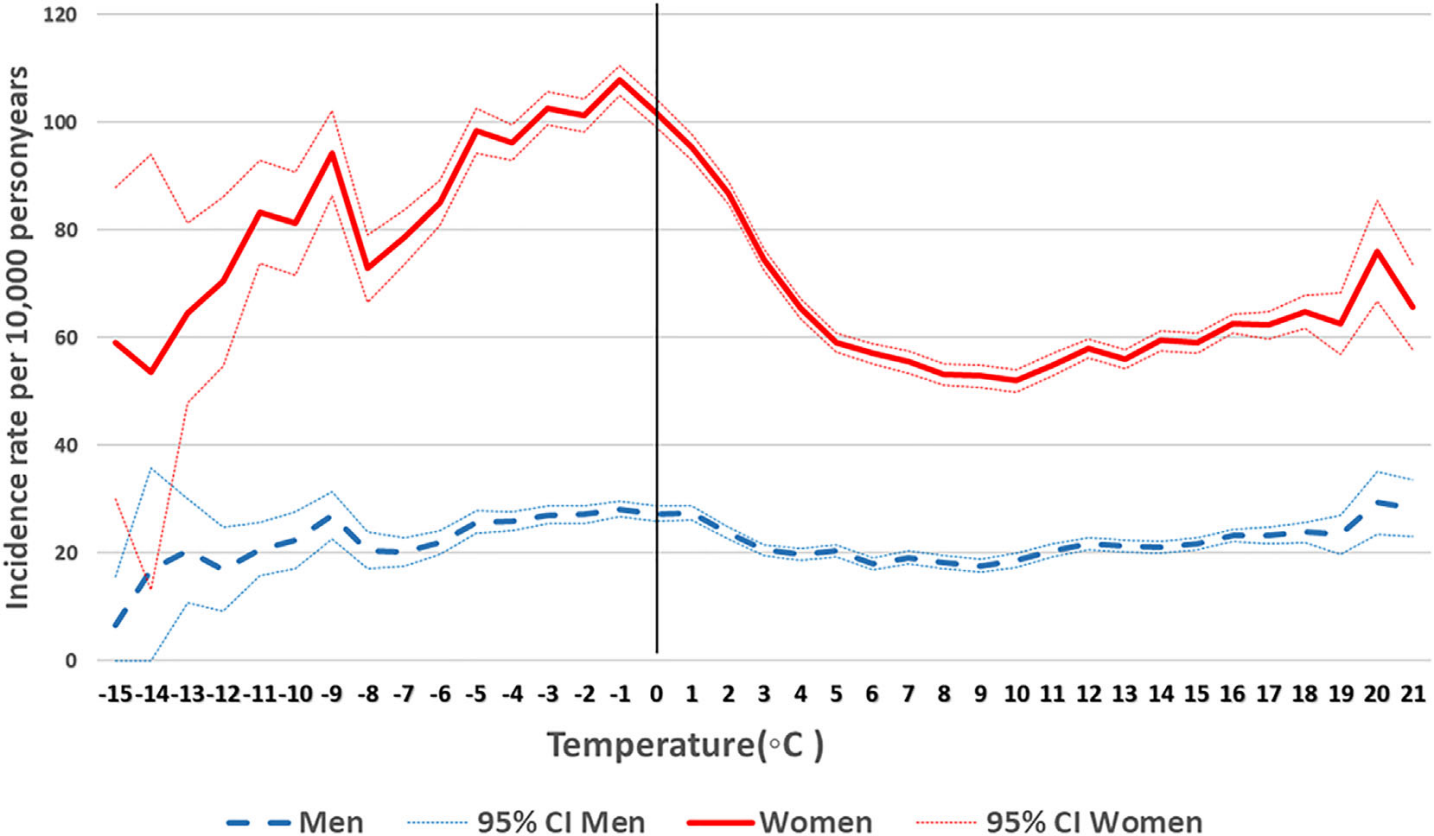
Age‐standardized incidence (95% CIs) of forearm fracture in women and men by average monthly temperature. Nationwide population (40–102 years) from 2008 to 2018.

**Fig. 2:**
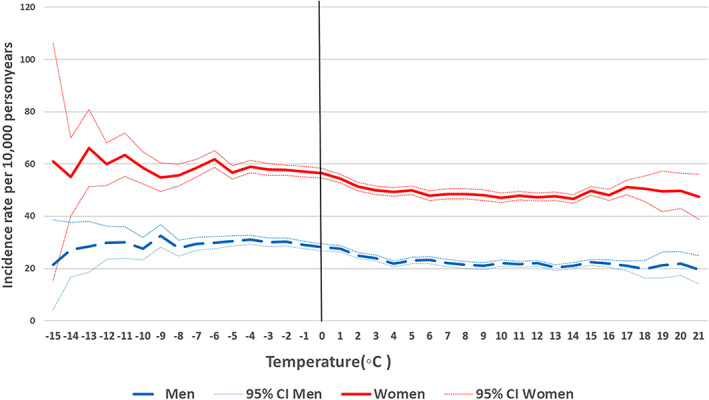
Age‐standardized incidence (95% CIs) of hip fracture in women and men by average monthly temperature. Nationwide population (50–102 years) from 2008 to 2018.

Figures 1 and 2 show the age‐standardized incidences of forearm and hip fracture over temperature. Stratified by gender, a 61% higher incidence of forearm fracture was found in women, and a 27% higher incidence in men (Table 2) at temperatures <0°C versus ≥0°C after adjustment. The rate difference based on age‐standardized incidences at cold versus warmer temperatures was also higher in women (36 fractures per 10,000 person‐years), compared with men (5.3 fractures per 10,000 person‐years). For both men and women, the highest incidence of forearm fracture was found at temperatures between −5°C and 0°C, and not in the coldest spectrum of <−5°C (Fig. 1, Table S1). The rate of forearm fracture at low temperatures was higher at all ages for both women and men (Fig. S2 *A*,*B*, *p* interaction age*temperature <0.001), and, for women, at <0°C there was a greater increase in rates when age increased (Fig. S2 *A*).

**Table 2:** Risk of Forearm Fracture (≥40 Years), and Hip Fracture (≥50 Years) With Cold (<0°C) Versus Warmer (≥0°C, Reference) Ambient Temperatures: Nationwide Population (Ages 40–102) From 2008–2018

	Temperature (°C)	Age‐standardized incidence^a^	IRR (95% CI)^b^	IRR (95% CI)^c^
Forearm fracture				
Women	≥0	62.4	Ref (−)	Ref (−)
	<0	98.4	1.58 (1.56–1.60)***	1.61 (1.59–1.63)***
Men	≥0	21.0	Ref (−)	Ref (−)
	<0	26.3	1.25 (1.22–1.28)***	1.27 (1.24–1.30)***
Hip fracture				
Women	≥0	49.3	Ref (−)	Ref (−)
	<0	58.0	1.18 (1.16–1.19)***	1.16 (1.14–1.18)***
Men	≥0	22.8	Ref (−)	Ref (−)
	<0	29.9	1.31 (1.28–1.34)***	1.31 (1.28–1.34)***

CI = confidence interval; IRR = incidence rate ratio.

^a^ Per 10,000 person years. Entire population >40 years (2008–2018) used as standard.

^b^ IRR (95% CI). Age adjusted.

^c^ IRR (95% CI). Adjusted for age, calendar year, health region of residence, urbanization degree, elevation, coastal proximity. Further adjustment for education level, marital‐ and immigrant status did not change the estimates.

*** *p* < 0.001.

There was also a gender difference in the IRR of hip fracture with temperature, with a stronger association in men compared with women, contrary to that of forearm fracture. The rate of hip fracture was 16% higher in women, and 31% higher in men at <0°C versus ≥0°C (Fig. 2, Table 2). Although the association in men was stronger on the relative scale, the rate difference in age standardized incidence was similar in both genders (7.1 fractures in men and 8.7 fractures in women per 10,000 person years). The hip fracture rate increased gradually with colder temperatures (Fig. 2, Table S1). When plotted against age, the rates for cold and warmer temperatures started to diverge between 70 and 80 years (Fig. S3 *A*,*B*, *p* interaction age*temperature <0.001), and differences increased further with age.

### Sensitivity analysis: additional adjustment for season and precipitation

Adjusting for season attenuated the associations between temperature and forearm fracture (IRR 1.27; 95% CI, 1.25–1.30) and hip fracture (IRR 1.08; 95% CI, 1.06–1.11) in women (not shown in tables), as compared to IRR 1.61 and IRR 1.16, respectively (as shown in Table 2). In men, the estimates were also attenuated: IRR forearm 1.19 (95% CI, 1.15–1.24) and IRR hip 1.13 (95% CI, 1.10–1.17) (not shown in tables), as compared to IRR forearm 1.27 and IRR hip 1.31. Associations between ambient temperature and fracture in both women and men were unchanged with further adjustment for precipitation (total mm/month).

### Post–hip fracture mortality with cold temperatures

**Fig. 3:**
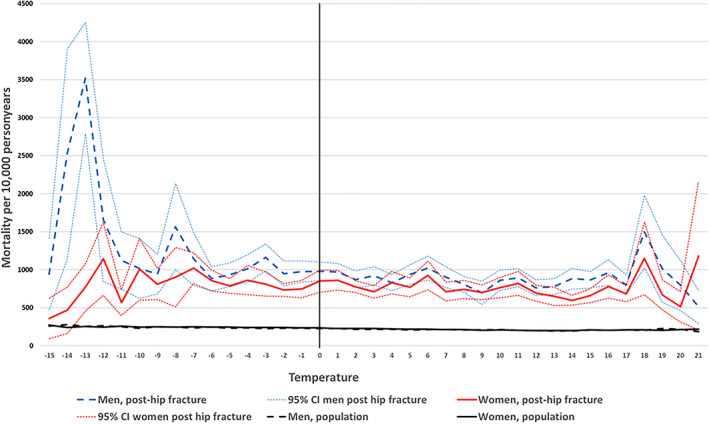
Age‐standardized all‐cause mortality (95% CIs) in the nationwide general population and in the post–hip fracture population (ages 50–102 years, from 2008 to 2018) by gender and average monthly temperature.

**Fig. 4:**
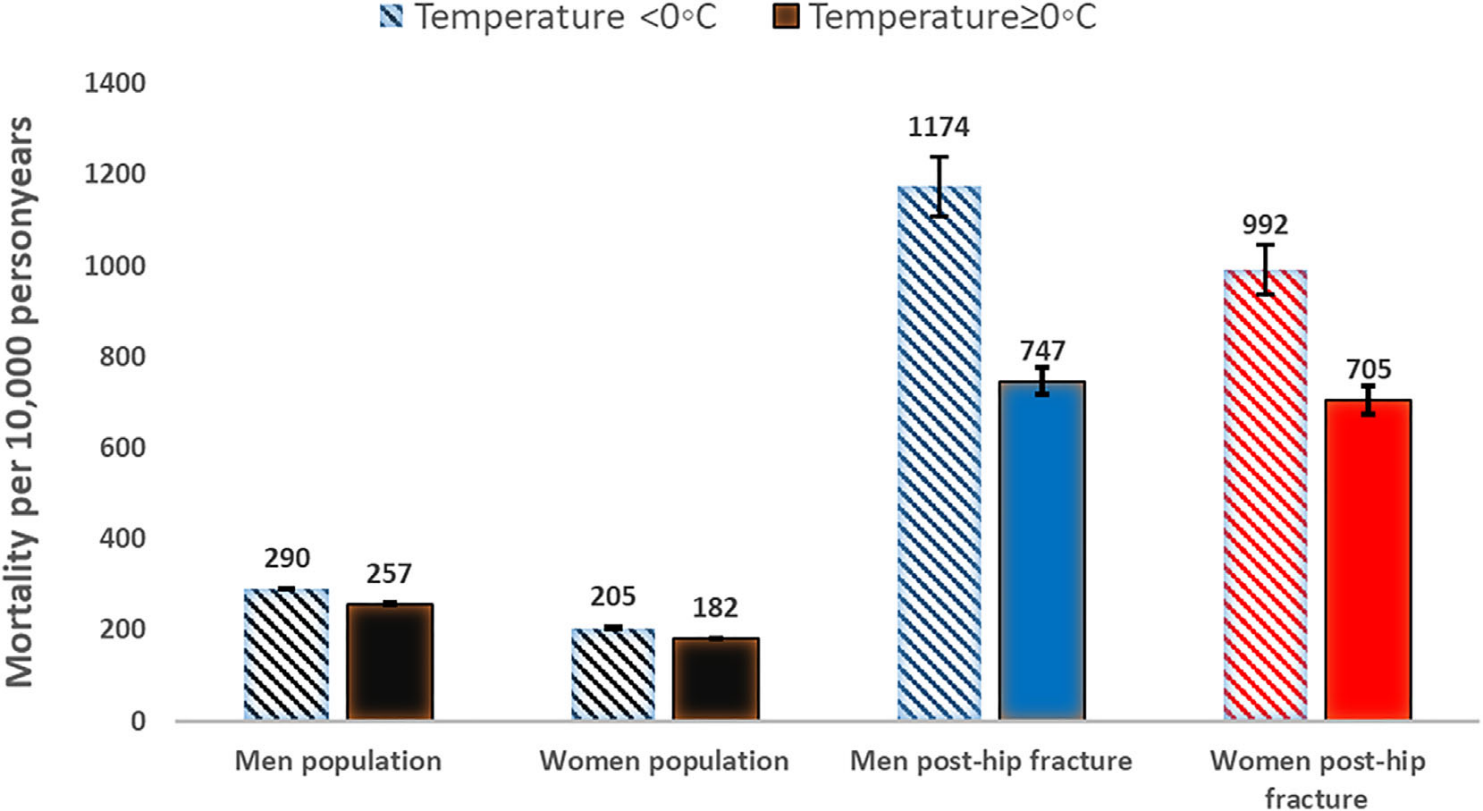
Age‐standardized all‐cause mortality (95% CIs) by temperature (<0°C and ≥0°C) and gender in the nationwide general population and in the post–hip fracture population (ages 50–102 years, from 2008 to 2018).

In the hip fracture patients, a higher all‐cause mortality was found in months with low temperatures (<0°C) compared with months with temperatures ≥0°C (HR women 1.13; 95% CI, 1.11–1.16; HR men 1.11; 95% CI, 1.07–1.15). The differences in mortality by temperature were greater in the post hip fracture population compared to the nationwide general population (Figs. 3 and 4 showing age standardized incidences by temperature). When taking the monthly population mortality rate into account, the excess mortality was 8% higher in men (HR 1.08; 95% CI, 1.02–1.14) and 9% higher in women (HR 1.09; 95% CI, 1.04–1.14). Table 3 gives the relative increased risk in mortality after hip fracture by follow‐up time and gender. The largest increase in risk by temperature was found with long‐term mortality >1 years. At >1 years follow up there was a trend of increasing mortality with colder temperatures (Table S2). The difference in relative survival by temperature did not vary by age (Fig. S4).

**Table 3:** Post–Hip Fracture Mortality With Cold (<0°C) Versus Warmer (≥0°C, Reference) Ambient Temperature. Relative Mortality (Association) Shown by Follow‐Up Time and Gender: Nationwide Hip Fracture Population (Ages 50–102 Years) From 2008–2018

Gender	Temperature (°C)	Age‐standardized mortality^a^	HR (95% CI)^b^ ≤3 months	HR (95% CI)^b^ >3–12 months	HR (95% CI)^b^ >1–11 years
Women	≥0	757	Ref (−)	Ref (−)	Ref (−)
	<0	803	0.99 (0.92–1.06)	1.02 (0.90–1.15)	1.26 (1.17–1.34)***
Men	≥0	884	Ref (−)	Ref (−)	Ref (−)
	<0	1054	1.02 (0.95–1.10)	1.17 (1.03–1.33)*	1.16 (1.06–1.27)**

CI = confidence interval; HR = hazard ratio.

^a^ Per 10,000 person years. Entire hip fracture population >50 years (2008–2018) used as standard.

^b^ HR (95% CI). Adjusted for age, calendar year, health region of residence, urbanization degree, elevation and coastal proximity. Further adjustment for education level, marital, and immigrant status did not change the estimates. Population mortality in the same month taken into account.

* *p* < 0.05.

** *p* < 0.01.

*** *p* < 0.001.

When stratifying on CCI, ie, comparing the post hip fracture mortality at <0°C versus ≥0°C *within* comorbidity levels, the relative excess mortality from colder temperature was most prominent in the group with no registered comorbidity: an 81% higher mortality in women (HR 1.81; 95% CI, 1.65–1.99), and 66% higher mortality in men (HR 1.66; 95% CI, 1.49–1.86). There was no excess post–hip fracture mortality at cold temperatures in the groups with registered comorbid diagnoses (Table S3).

## Discussion

In this longitudinal, register‐based study, which included the entire population of Norway over 40 years from 2008 to 2018, we found a higher risk of both forearm and hip fracture at temperatures <0°C. The increase in risk with cold temperatures differed by fracture type, gender, and age. The greatest relative risk of forearm fracture was at temperatures just below freezing (0°C), in women, and in older age, whereas the relative risk of hip fracture increased gradually with increasingly colder temperatures, was greatest in men, and in older age. There was also a higher mortality in hip fracture patients at cold temperatures, even when taking into account the underlying mortality in the population. This excess mortality associated with temperature was particularly high in the hip fracture patients without comorbidity.

### Forearm fracture

The large variations in ambient temperature, ranging from an average monthly temperature of −20°C to 22°C throughout the year makes Norway an ideal place to study its influence on osteoporotic fractures and post–hip fracture mortality. We found a strong association between temperature and risk of forearm fractures, and the association was stronger in women than in men (61% versus 27% higher incidence). Surges of forearm fractures are commonly reported by the emergency units during wintertime; however, this is the first time the increase in risk has been estimated on a national level. Previous Norwegian studies looking at seasonal variation and risk of forearm fracture have been small, and from limited geographic areas.^(^ ^7^, ^11^, ^12^, ^43^ ^)^ In line with our results, two of these studies found the risk increase during wintertime to be greatest in women.^(^ ^11^, ^12^ ^)^ However, other studies only found seasonal variations in fractures occurring outdoors,^(^ ^7^ ^)^ or in high‐energy distal forearm fractures.^(^ ^43^ ^)^ Studies from the UK and the United States have also found an increase in forearm fractures with cold temperatures.^(^ ^10^, ^13^ ^)^ Similar to our results, a study by Johnson and colleagues^(^ ^6^ ^)^ found a greater difference in forearm fracture incidence associated with temperature in women than in men, but with the greatest differences in the younger age group (40–69 years).^(^ ^6^ ^)^ In a sample from the US Medicare population, winter peaks in fracture, particularly distal forearm fractures were higher in the men ≤80 years.^(^ ^13^ ^)^ This was contrary to our study, were the relative risk increased with age. Because most distal forearm fractures have been found to occur outdoors,^(^ ^6^, ^13^ ^)^ these differences could indicate cultural gender and age variations in time spent outdoors when temperatures are cold, and possibly a more active older population due to better physical capacity in recent years.^(^ ^44^ ^)^

### Hip fracture

We found a higher risk of hip fracture with colder temperatures, although not as high as for forearm fracture. This is in agreement with previous studies, where a smaller seasonal variation in hip fractures has been found compared to seasonal variation in risk of other types of fractures. In a systematic review of the association between climate and hip fractures, Roman Ortiz and colleagues^(^ ^19^ ^)^ summarized 20 studies, of which 19 had ecological design and one had a case‐crossover design. Most, but not all studies found an increase in hip fracture risk associated with low temperatures.^(^ ^45^ ^)^ We found a greater relative risk increase of hip fracture associated with cold temperatures in men compared to in women. In a study, from the UK, Johnson and colleagues^(^ ^10^ ^)^ also found the most prominent association between low temperature and increased hip fracture risk in men (>50 years). The difference in relative risk between women and men is intriguing and may suggest different gender mechanisms for the temperature‐fracture association.

### Mortality

Concurrent with the well‐documented population trend with higher winter mortality,^(^ ^1^, ^34^ ^)^ we found a higher mortality in hip fracture patients at cold temperatures. Although health impacts from cold weather are commonly seen globally (even with mild winters),^(^ ^34^ ^)^ post–hip fracture mortality according to ambient temperature has not been widely studied. Using aggregated hospital data, a national study in the UK found a higher 30‐day mortality (30.5% higher) among those presenting with hip fracture in the winter months of December to February compared with the rest of the year.^(^ ^21^ ^)^ Our data were not suitable for estimating mortality at 30 days, because deaths were recorded on a monthly basis; however, when investigating mortality at 3 months and long‐term follow‐up, we found the greatest excess mortality at long‐term follow‐up. Because temperature was measured in the month of death, this may indicate a long‐lasting vulnerability in the hip fracture patients. Interestingly, it was the hip fracture group with no underlying comorbidity that had the greatest relative mortality by temperature. This could reflect a higher activity‐level during wintertime of this presumably healthy patient group, with more exposure to variations in outdoor temperature.

### Potential mechanisms and prevention

Studies of the short‐term effect of temperature suggest the higher risk of forearm fracture at colder temperatures is due to slippery and icy conditions outdoors, produced by an abrupt change in temperature along with precipitation.^(^ ^10^, ^13^ ^)^ This hypothesis is supported by our finding of a particularly high incidence of forearm fractures at temperatures between −5°C and 0°C, which is the spectrum when rainfall often freezes on the ground, creating black ice. Cold temperatures have also been associated with other acute fall‐related incidences, such as hip fractures and deaths in a systematic review.^(^ ^46^ ^)^ The authors stated that exposure to cold may cause a loss of coordination and lengthen reaction time, and extra layers of clothing may limit mobility, thus making older adults more prone to falling.^(^ ^46^ ^)^ Other factors not directly related to temperature, such as low visibility due to darkness during the winter months may also be important.

In the long‐term, cold temperatures may reduce physical activity and outdoor exposure to vitamin D from daylight, which could lead to bone fragility and increase the risk of fracture.^(^ ^19^ ^)^ Due to variations in sunlight, vitamin D production in the skin varies across Norway. However, in coastal regions of Northern Norway, the low ultraviolet B (UVB) availability is compensated for by strong habits of cod liver oil supplementation and fatty fish consumption,^(^ ^47^ ^)^ and may not be as strongly correlated with temperature as in other populations.

Health impacts and high mortality during months with cold temperatures may also be due to underlying cardiovascular and respiratory problems, and possibly seasonal peaks in influenza.^(^ ^34^, ^48^, ^49^ ^)^ In women, the strikingly high age‐standardized incidence of forearm fractures at temperatures just below freezing (102/10,000 person‐years at −5°C to 0°C, versus 58/10,000 person‐years at 0°C to 5°C) amounted to a surplus of 45 fractures per 10,000 person‐years, whereas in men this number was only six per 10,000 person‐years. These differences signify the substantial potential for prevention; however, greater understanding of the underlying mechanisms is needed.

### Strengths and limitations

Strengths of our study include an almost complete capture of forearm and hip fractures and demographic information on all Norwegian residents over a long period of time, which gave very precise estimates of hip fracture incidence. However, whereas nearly all hip fractures are treated at hospitals, this is not the case for forearm fractures, which could lead to less precise reporting. We did not have access to forearm fractures treated only in primary care. Although data from an ongoing study show that this proportion is low (approximately 6%), it may be a limitation if the fracture‐reporting also varies by mean temperature of the geographic location. As opposed to many previous studies on temperature and fracture incidence, we had individual level fracture data, and national data on ambient temperature was measured within small geographic units (smaller than postal codes). We were also able to adjust for many potential confounders, and we had enough statistical power to stratify on gender and age. For the first time, the excess all‐cause post–hip fracture mortality in association with outdoor temperatures was estimated on an individual level, taking the underlying population mortality into account. Using average monthly temperature in the current study gave the opportunity to see effects of both acute events (ie, falls due to icy conditions), and also possible biological changes in the body due to persistent cold. Still, it would have been an advantage to be able to separate the two effects. We also did not have information on where the fracture occurred, and there may be misclassification of the exposure if the fracture occurred somewhere other than at the place of residence; however, we expect this error to be small. Our data do not allow examination of underlying mechanisms for the temperature‐fracture and temperature‐mortality associations. Data separating indoor and outdoor fractures would have been useful to examine mechanisms of the gender and age differences, as would data on vitamin D supplements. Variations may be related to external factors, such as the risk of falling due to snow and ice on the ground. We did not find that variations in precipitation had an impact on the association between temperature and fracture; however, we had no information on whether the precipitation stayed on the ground as snow or as ice. Variations may also be due to internal factors such as differences in bone mineral density, muscle strength, and other factors related to lifestyle (eg, diet, body mass index, and physical activity) and medication use. Comorbidity was estimated by CCI, which is based on a predefined set of hospital‐registered chronic diseases during the year of the fracture. It is therefore possible that these patients had underlying comorbid diagnoses that were not severe enough to be recorded at the time of admittance.

## Conclusion

We found a higher incidence of both forearm and hip fracture with cold ambient temperatures, with stronger associations in older age, which differed by fracture type and gender. We present new information on excess mortality in hip fracture patients at cold temperatures. Even when taking into account a higher winter‐mortality in the background population, there was an increased risk of all‐cause mortality in hip fracture patients at low ambient temperatures. This risk increase was greatest at long‐term follow‐up and in the healthiest patients. Possible risk factors for excess mortality with cold temperatures, and the temperature‐associated differences in fracture incidence by gender and age, should be studied further.

## Author Contributions

**Cecilie Dahl:** Conceptualization; data curation; formal analysis; funding acquisition; investigation; methodology; project administration; validation; visualization; writing – original draft; writing – review and editing. **Christian Madsen:** Data curation; investigation; methodology; software; writing – review and editing. **Tone Kristin Omsland:** Writing – review and editing. **Anne Johanne Soslashgaard:** Writing – review and editing. **Ketil Tunheim:** Data curation; writing – review and editing. **Hein Stigum:** Methodology; writing – review and editing. **Kristin Holvik:** Methodology; writing – review and editing. **Haakon E Meyer:** Conceptualization; data curation; funding acquisition; methodology; supervision; writing – review and editing.

## Disclaimer

Data from the Norwegian Patient Registry has been used in this publication. The interpretation and reporting of these data are the sole responsibility of the authors, and no endorsement by the Norwegian Patient Registry is intended or should be inferred.

## Conflict of Interest

All authors state no conflict of interest.

### Peer Review

The peer review history for this article is available at https://publons.com/publon/10.1002/jbmr.4628.

## Supporting information


Table S1
Table S2
Table S3

Fig. S1
Fig. S2
Fig. S3
Fig. S4

## Data Availability

Data available on request due to privacy/ethical restrictions
