# Supplementary material for: The Association of Cold Ambient Temperature With Fracture Risk and Mortality: National Data From Norway—A Norwegian Epidemiologic Osteoporosis Studies (NOREPOS) Study
Source: J Bone Miner Res. 2022 Jun 25;37(8):1527–36. doi: 10.1002/jbmr.4628 (PMC9545665; doi:10.1002/jbmr.4628)
Supplement: Supplementary file 1 — Table S1 Table S2 Table S3 [file JBMR-37-1527-s002.docx]

**Supplementary tables**

*Fracture incidence*

**Supplementary table 1.** Risk of forearm fracture (≥40 years), and hip fracture (≥50 years) with categories of cold (-20 to -5^◦^C, -5 to 0^◦^C, and 0 to 5^◦^C), versus warmer ambient temperature >5 ◦C (reference). Nationwide population (ages 40-102) from 2008-2018.

|  |  | Temperature (^◦^C) | Age-standardized incidence^a^ | IRR (95% CI)^b^ | IRR (95% CI)^c^ |
| --- | --- | --- | --- | --- | --- |
| Forearm fracture | Women | >5 | 57.6 | Ref (-) | Ref (-) |
|  |  | 0-5 | 72.5 | 1.26 (1.24, 1.27)*** | 1.26 (1.24, 1.28)*** |
|  |  | -5 <0 | 102.4 | 1.78 (1.75, 1.80)*** | 1.80 (1.77, 1.83)*** |
|  |  | <-5 | 87.8 | 1.53 (1.49, 1.56)*** | 1.60 (1.55, 1.64)*** |
|  | Men | >5 | 20.9 | Ref (-) | Ref (-) |
|  |  | 0-5 | 21.4 | 1.03 (1.00, 1.05) | 1.03 (1.00, 1.06)* |
|  |  | -5 <0 | 27.2 | 1.31 (1.27, 1.34) *** | 1.33 (1.29, 1.37)*** |
|  |  | <-5 | 23.5 | 1.13 (1.08, 1.18)*** | 1.17 (1.11, 1.23)*** |
| Hip fracture | Women | >5 | 48.3 | Ref (-) | Ref (-) |
|  |  | 0-5 | 51.7 | 1.07 (1.05,1.09) *** | 1.08 (1.06, 1.10)*** |
|  |  | -5 <0 | 57.7 | 1.20 (1.18,1.22) *** | 1.19 (1.17, 1.22)*** |
|  |  | <-5 | 58.6 | 1.23 (1.19,1.26) *** | 1.18(1.14, 1.22)*** |
|  | Men | >5 | 21.7 | Ref (-) | Ref (-) |
|  |  | 0-5 | 25.0 | 1.15 (1.12,1.18)*** | 1.15 (1.12, 1.18)*** |
|  |  | -5 <0 | 29.8 | 1.37 (1.33,1.41)*** | 1.37 (1.33, 1.41)*** |
|  |  | <-5 | 30.0 | 1.38 (1.33, 1.44)*** | 1.37 (1.31, 1.43)*** |
| ****p<0.001*p<0.05*  a per 10,000 personyears. Entire population ≥40 years (2008-2018) used as standard  b Incidence rate ratio (95% Confidence Interval), age adjusted  c Incidence rate ratio (95% Confidence Interval), adjusted for age, calendar year, health region of residence, urbanization degree, elevation, coastal proximity. Additional adjustment for education level, marital- and immigrant-status did not change the estimates. | | | | | |

*Mortality*

**Supplementary table 2.** Post-hip fracture mortality with categories of cold (-20 to -5^◦^C, -5 to 0^◦^C, and 0 to 5^◦^C), versus warmer ambient temperature >5^◦^C (reference). Analyses stratified by follow-up time after fracture (at 1 year and 1-11 years). Nationwide population (50-102 years) from 2008-2018.

|  | Temperature (^◦^C) | Age-standardized mortality^a^ | HR (95% CI)^b^  ≤12 months | HR (95% CI)^b^  >1-11 years |
| --- | --- | --- | --- | --- |
| Women | >5 | 735 | Ref (-) | Ref (-) |
|  | 0-5 | 807 | 1.05 (0.99, 1.12) | 1.20 (1.12, 1.29)*** |
|  | -5 <0 | 778 | 1.00 (0.93, 1.07) | 1.29 (1.19, 1.40)*** |
|  | <-5 | 871 | 1.01 (0.91, 1.12) | 1.46 (1.30, 1.65)*** |
| Men | >5 | 870 | Ref (-) | Ref (-) |
|  | 0-5 | 916 | 1.00 (0.94, 1.07) | 1.16 (1.06, 1.27)** |
|  | -5 <0 | 1006 | 1.02 (0.95, 1.10) | 1.20 (1.08, 1.34)** |
|  | <-5 | 1169 | 1.12 (1.01, 1.25)* | 1.27 (1.07, 1.50)** |
| ****p<0.001, **p<0.01, *p<0.05*  a per 10,000 personyears. Entire population >50 years (2008-2018) used as standard  b Hazard ratio (95% Confidence Interval), adjusted for age, calendar year, health region of residence, urbanization degree, elevation, coastal proximity, and underlying population mortality. Further adjustment for education level, marital- and immigrant-status did not change the estimates. | | | | |

**Supplementary table 3.** Absolute and relative post hip fracture mortality at cold (<0^◦^C) versus warmer ambient temperature ≥0^◦^C (reference), stratified on Charlson Comorbidity Index based on registered diagnoses in the year of the fracture. Nationwide population (50-102 years) from 2008-2018.

|  | Charlson group | Temperature (^◦^C) | Age-standardized mortality^a^ | HR (95% CI)^b^ |
| --- | --- | --- | --- | --- |
| Women | 0 (No comorbidity) | ≥0 | 363 | Ref (-) |
|  |  | <0 | 512 | 1.81 (1.65, 1.99)*** |
|  | 1 (index 1) | ≥0 | 812 | Ref (-) |
|  |  | <0 | 882 | 1.01 (0.92, 1.11) |
|  | 2 (index 2-15) | ≥0 | 2314 | Ref (-) |
|  |  | <0 | 2008 | 0.86 (0.81, 0.92)*** |
| Men | 0 (No comorbidity) | ≥0 | 407 | Ref (-) |
|  |  | <0 | 625 | 1.66 (1.49, 1.86)*** |
|  | 1 (index 1) | ≥0 | 992 | Ref (-) |
|  |  | <0 | 1068 | 0.99 (0.87, 1.11) |
|  | 2 (index 2-15) | ≥0 | 2296 | Ref (-) |
|  |  | <0 | 2479 | 0.96 (0.90, 1.03) |
| ****p<0.001, **p<0.01, *p<0.05*  a per 10,000 personyears. Entire population ≥50 years (2008-2018) used as standard  b Hazard ratio (95% Confidence Interval), adjusted for age, calendar year, health region of residence, urbanization degree, elevation, coastal proximity and population mortality. Further adjustment for education level, marital status and immigrant status did not change the estimates. | | | | |
