# Supplementary material for: The Association of Cold Ambient Temperature With Fracture Risk and Mortality: National Data From Norway—A Norwegian Epidemiologic Osteoporosis Studies (NOREPOS) Study
Source: J Bone Miner Res. 2022 Jun 25;37(8):1527–36. doi: 10.1002/jbmr.4628 (PMC9545665; doi:10.1002/jbmr.4628)
Supplement: Supplementary file 2 — Fig. S1 Fig. S2 Fig. S3 Fig. S4 [file JBMR-37-1527-s001.docx]

**Supplementary figures**


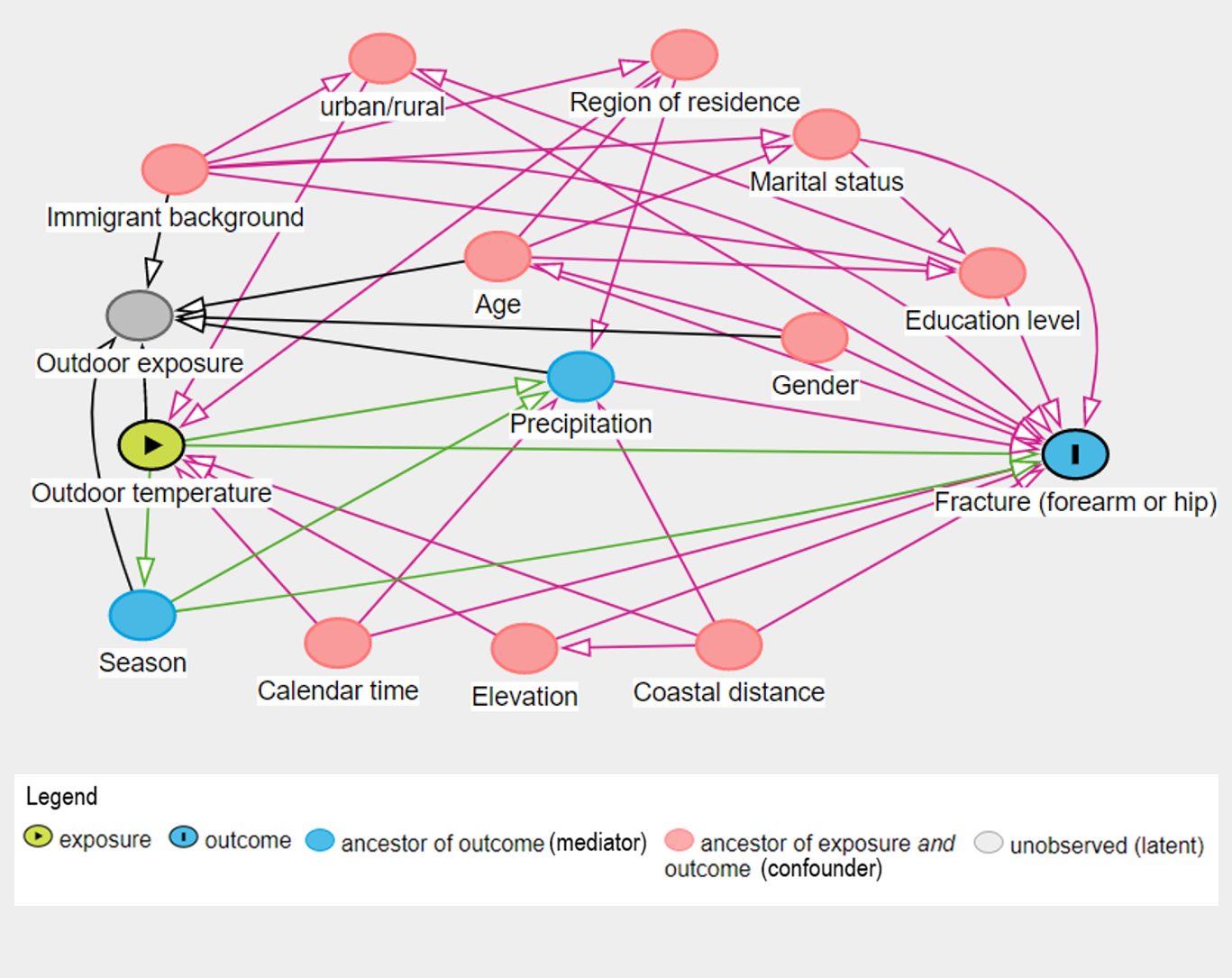


**Supplementary figure 1a**. Directed Acyclic Graph (DAG) of the proposed causal relation between outdoor temperature and fracture (forearm or hip). Green arrows= causal pathways, red arrows= biasing pathways. Minimum sufficient adjustment set for the total effect: calendar time, coastal distance, elevation, region of residence, urban/rural residence.

DAGs were constructed using the daggity tool. Reference: Textor J, van der Zander B, Gilthorpe MS, Liskiewicz M, Ellison GT. Robust causal inference using directed acyclic graphs: the R package 'dagitty'. Int J Epidemiol. Dec 1 2016;45(6):1887-94. Epub 2017/01/17.


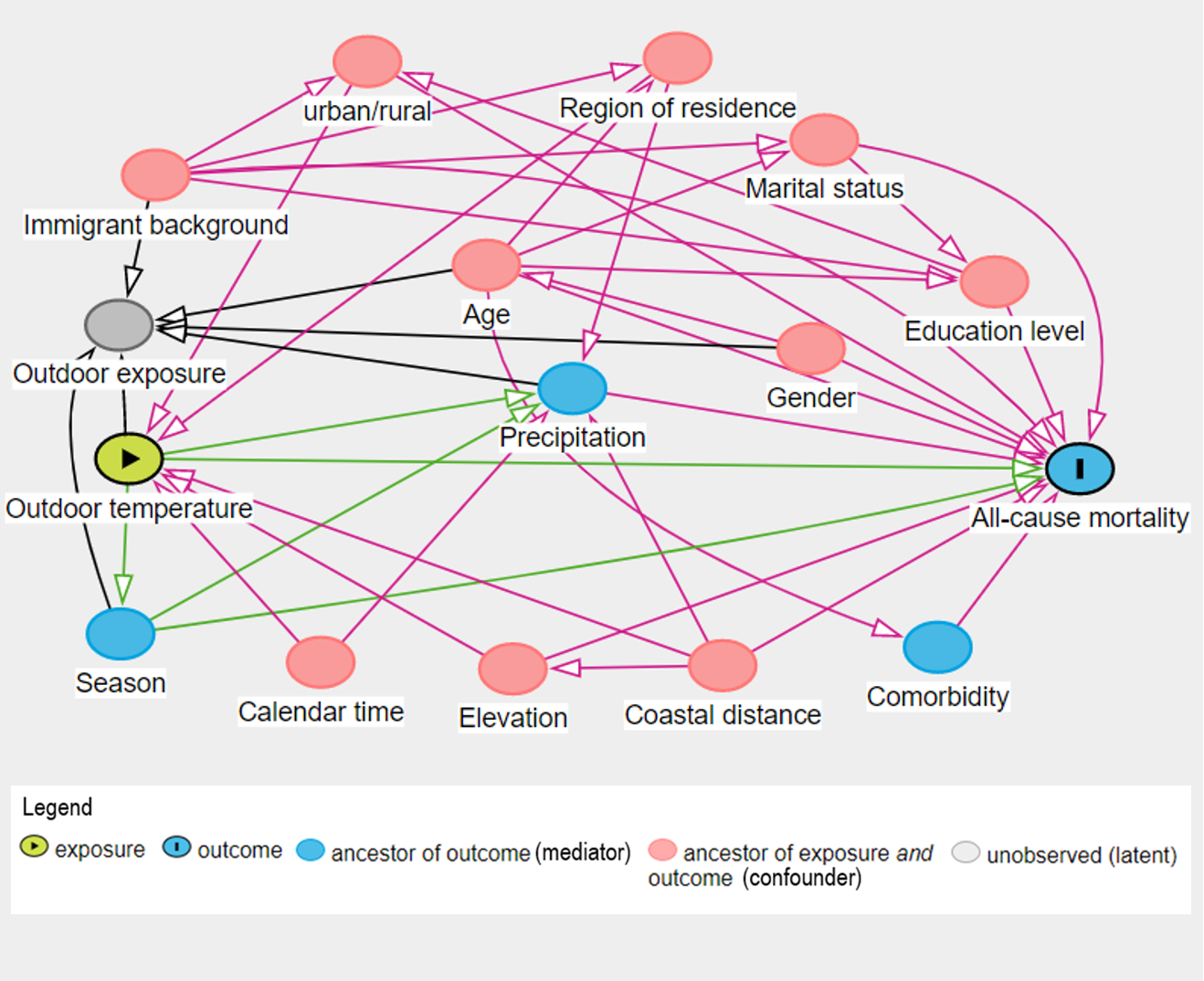


**Supplementary figure 1b**. Directed Acyclic Graph (DAG) of the proposed causal relation between outdoor temperature and all-cause mortality in hip fracture patients. Green arrows= causal pathways, red arrows= biasing pathways. Minimum sufficient adjustment set for the total effect: calendar time, coastal distance, elevation, region of residence, urban/rural residence.

DAGs were constructed using the daggity tool. Reference: Textor J, van der Zander B, Gilthorpe MS, Liskiewicz M, Ellison GT. Robust causal inference using directed acyclic graphs: the R package 'dagitty'. Int J Epidemiol. Dec 1 2016;45(6):1887-94. Epub 2017/01/17.


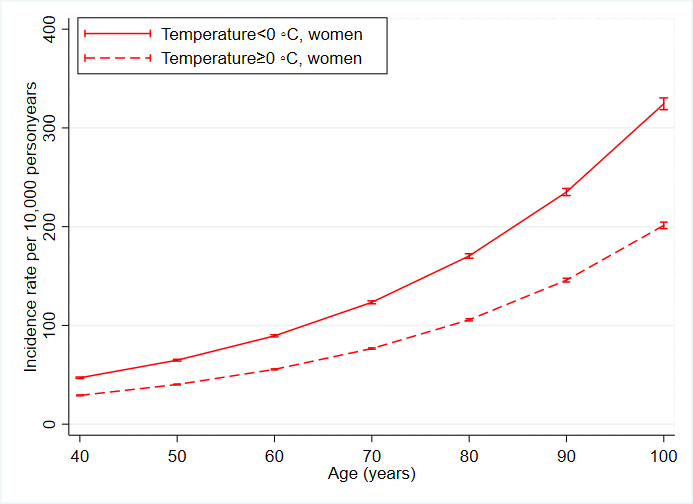


**Supplementary figure 2a**. Predicted incidence rate (95% CI) of forearm fractures per 10,000 person years in women over age, stratified by low temperature (solid line) and high temperature (dashed line) and adjusted for calendar year, region of residence, urban/rural residence and geography (residential elevation and coastal proximity). Nationwide population (ages 40-102) from 2008-2018.


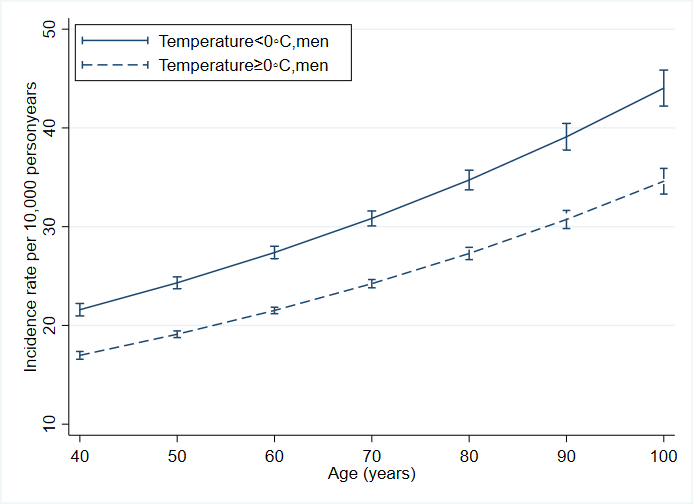


**Supplementary figure 2b.** Predicted incidence rate (95% CI) of forearm fractures per 10,000 person years in men over age, stratified by low temperature (solid line) and high temperature (dashed line) and adjusted for calendar year, region of residence, urban/rural residence and geography (residential elevation and coastal proximity). Nationwide population (ages 40-102) from 2008-2018.


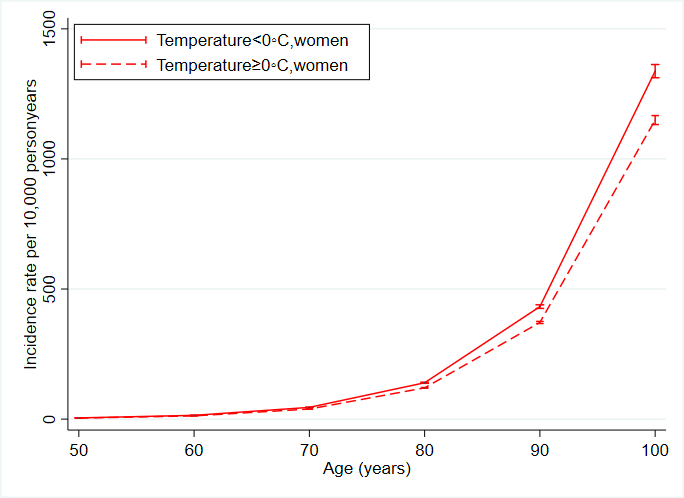


**Supplementary figure 3a**. Predicted incidence rate (95% CI) of hip fractures per 10,000 person years in women over age, stratified by low temperature (solid line) and high temperature (dashed line) and adjusted for calendar year, region of residence, urban/rural residence and geography (residential elevation and coastal proximity). Nationwide population (ages 50-102) from 2008-2018.


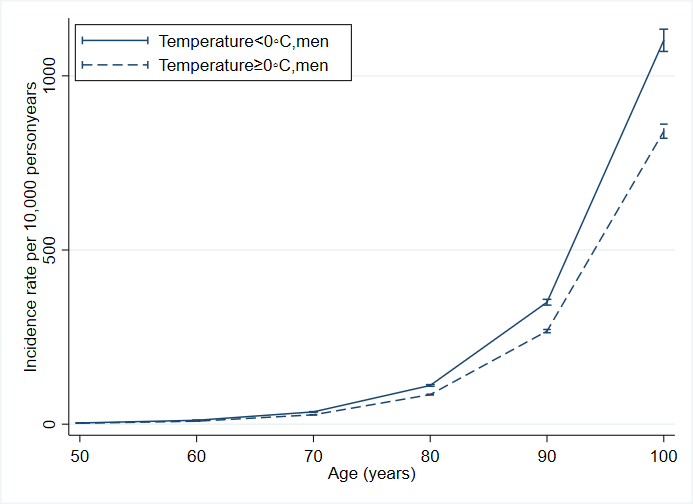


**Supplementary figure 3b**. Predicted incidence rate (95% CI) of hip fractures per 10,000 person years in men over age, stratified by low temperature (solid line) and high temperature (dashed line) and adjusted for calendar year, region of residence, urban/rural residence and geography (residential elevation and coastal proximity). Nationwide population (ages 50-102) from 2008-2018.


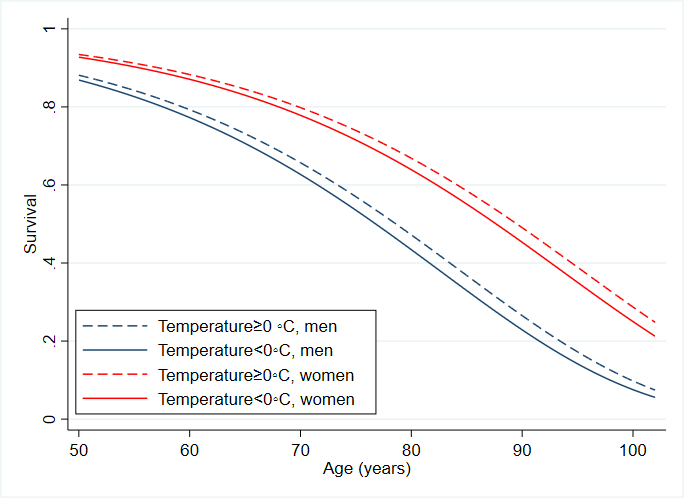


**Supplementary figure 4.** Predicted relative survival in the hip fracture population across age and stratified by low temperature (solid line) and high temperature (dashed line) and gender (men in blue and women in red). Nationwide population (ages 50-102) from 2008-2018.

The survival is predicted across the age span (age at death) from a Flexible parametric survival model that adjusts for gender and calendar time. The model also takes into account the mortality in the background population at the same age (i.e. relative survival), using the bhazard option in Stata.
